# Upper gastrointestinal bleeding from primary aortoesophageal fistula in a patient with aneurism of the thoracoabdominal aorta: Case report and literature review

**DOI:** 10.1002/ccr3.9311

**Published:** 2024-08-15

**Authors:** Elena Curakova Ristovska, Gregor Krstevski, Misel Andov, Aleksandar Kolev, Kristijan Bundovski, Kemal Rusiti, Svetozar Antovic, Ivan Rankovic, Smiljana Bundovska Kocev, Natasa Hadzi‐Nikolova Alcinova, Ante Bogut

**Affiliations:** ^1^ University Clinic for Gastroenterohepathology Skopje Republic of North Macedonia; ^2^ Faculty of Medicine Ss. Cyril and Methodius University in Skopje Skopje Republic of North Macedonia; ^3^ University Clinic of Thoracic and Vascular Surgery Skopje Republic of North Macedonia; ^4^ University Clinic for State Cardiac Surgery Skopje Republic of North Macedonia; ^5^ University Clinic of Digestive surgery Skopje Republic of North Macedonia; ^6^ Department of Gastroenterology and Liver Unit Royal Cornwall Hospitals NHS trust Truro England UK; ^7^ University School of Medicine Peninsula NHS Education London England Southwest UK; ^8^ University Clinic of Radiology Skopje Republic of North Macedonia; ^9^ School of Medicine, University of Mostar Mostar Bosnia and Herzegovina; ^10^ Department of Gastroenterology University Hospital Mostar Mostar Bosnia and Herzegovina

**Keywords:** aortic aneurysm, computed tomography, gastroscopy, primary aortoesophageal fistula, upper gastrointestinal bleeding

## Abstract

**Key Clinical Message:**

Upper gastrointestinal bleeding due to primary aortoesophageal fistula is a rare clinical condition burdened with high mortality rate. However, the outcomes are closely related to the level of clinical awareness, the complementary and multidisciplinary approach during the diagnostic workup, and the selected treatment option.

**Abstract:**

We present an atypical case of an aneurysm of the thoracoabdominal aorta complicated with primary aortoesophageal fistula (AEF). A 55‐year‐old male with no previous diseases, presented with prolonged and intense back pain and upper gastrointestinal bleeding. The gastroscopy detected an unusual culprit lesion in the distal esophagus resembling an esophageal wall defect, and the computed tomography revealed an aneurysm of the thoracoabdominal aorta, remarkable surrounding hematoma, and active contrast extravasation. Despite the urgent surgical repair, a lethal outcome occurred. AEF patients require high clinical awareness and complementary multidisciplinary approach in order to provide a rapid diagnosis and optimal treatment.

## 1 INTRODUCTION

Aortoesophageal fistula (AEF) is an abnormal communication between the aorta and the esophagus that represents approximately 10% of all the aortoenteric fistulas.^1^ AEF is a rare, but serious life‐threatening cause of upper gastrointestinal bleeding (UGB) burdened with a high mortality rate.^2^, ^3^, ^4^ Primary AEF occurs in patients with native aorta, while the secondary AEF occurs after surgery of the thoracic aorta or the esophagus. The estimated annual incidence of primary and secondary AEFs is about 0.0015% and 0.6%–2%, respectively.^5^ The primary AEF is mainly caused by atheromatous aortic aneurysms, or less frequently by penetrating aortic ulcer, tumor, foreign body, radiotherapy, or infection.^2^, ^6^, ^7^ The secondary AEF occurs due to infection of artificial blood vessels after artificial revascularization surgery.^8^ The entity was reported for the first time in 1818 by Dubrueil, while in 1914, Chiari described the typical clinical triad presented by sentinel bleeding, mid‐thoracic chest pain, and fatal exsanguinating hemorrhage after a variable symptom‐free interval.^7^ The gastroscopy usually detects the culprit bleeding lesion with unusual aspect, but the definitive diagnosis is made by the dynamic CT showing the aortic aneurysm and abnormal contrast extravasation. UGB due to AEF is burdened with a high morbidity and mortality rate, which raises the need for high clinical awareness and prompt diagnosis and treatment.

## 2 CASE HISTORY

We present an atypical case of male patient with an aneurysm of the thoracic and abdominal aorta complicated with UGB due to a primary AEF. A 55‐year‐old male patient was referred to our tertiary care gastroenterology center by a surgical emergency department due to hematemesis. A few days ago, the patient was prescribed NSAIDs and corticosteroids for a prolonged and severe back pain. There was no previous cardiovascular or gastrointestinal pathology whatsoever. On admission, he had stable vital signs (blood pressure, 140/90 mmHg; heart rate, 90/min; oxygenation, 98%), the initial blood count was slightly reduced (Hb, 141 g/L; RBC, 4.72 × 10^12^; HCT, 0.41; WBC, 23.3 × 10^9^; PLT, 326 × 10^9^), and BUN (9.2 μmol/L) and creatinine (103 μmol/L) were slightly elevated. After the initial evaluation, the patient was admitted to the Clinic for Gastroenterohepatology.

## 3 METHODS (DIFFERENTIAL DIAGNOSIS, INVESTIGATIONS AND TREATMENT)

**FIGURE 1:**
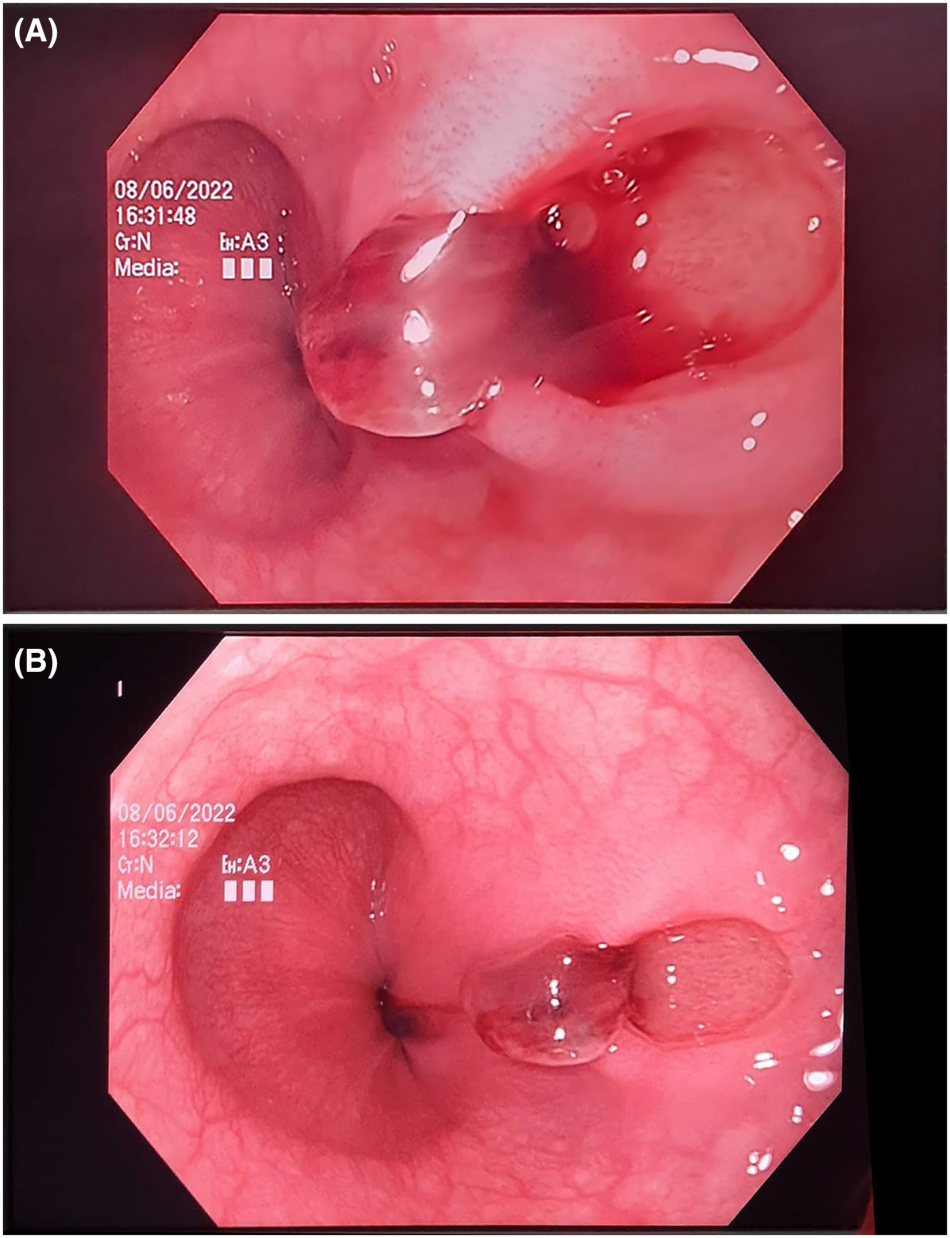
(A) Endoscopic aspect of the aortoesophageal fistula: Regular, oval mucosal lesion in distal esophagus, 7–8 mm in size, with a small adherent clot. (B) Endoscopic aspect of the aortoesophageal fistula: regular, oval mucosal lesion in distal esophagus, 7–8 mm in size.

**FIGURE 2:**
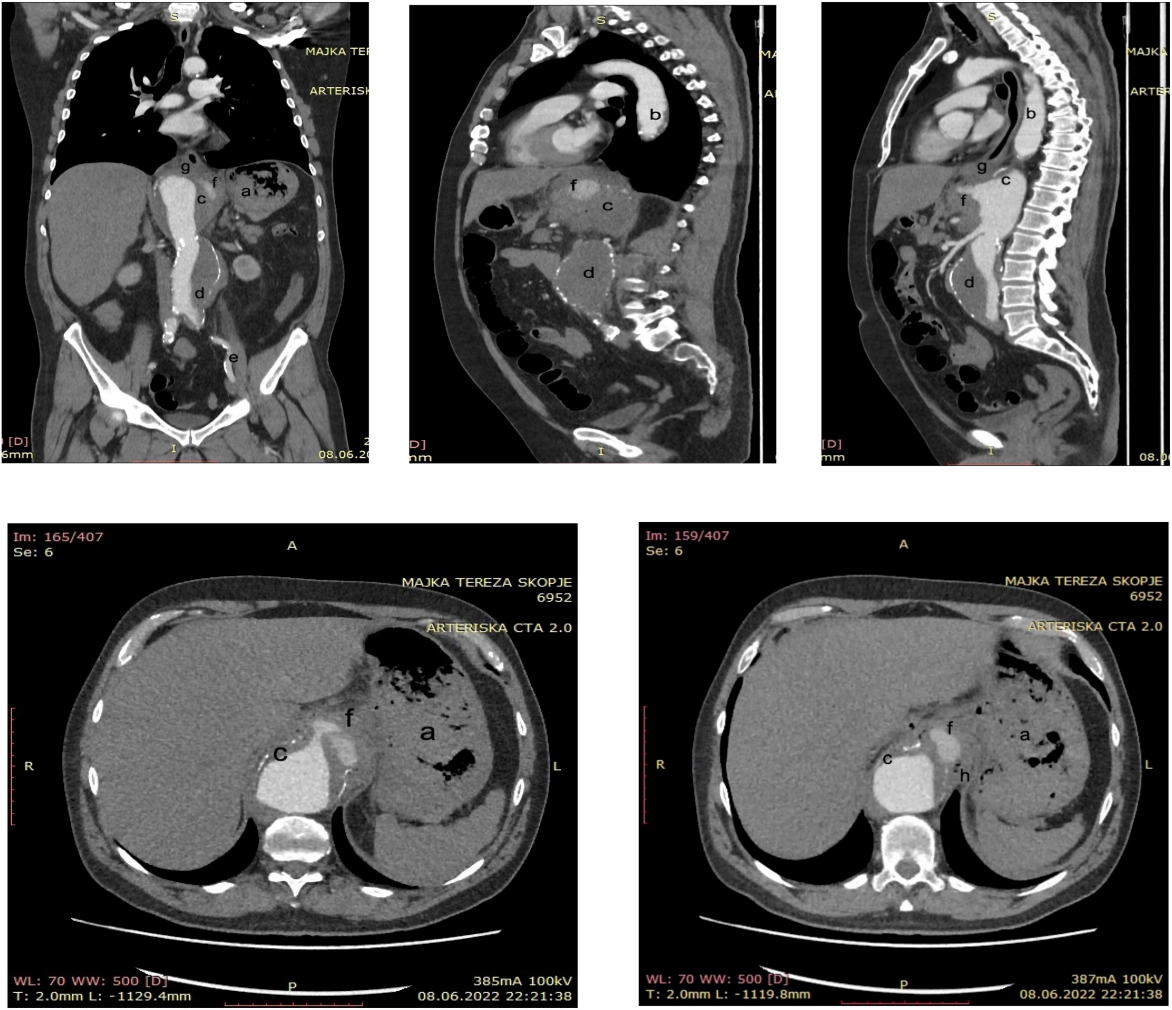
Thoracic and abdominal CT scan presenting the aneurysm of the thoracic and abdominal aorta propagating toward the left common iliac artery, mural thrombosis, and active contrast extravasation with air inclusions in the mediolateral segment of the extravasation. (a) Gastric content, (b) aneurism of the thoracic aorta, (c) suprarenal abdominal aorta, (d) infrarenal abdominal aorta, (e) aneurism propagating to the left common iliac artery, (f) contrast extravasation, (g) distal esophagus, and (h) air bubbles.

After the admission and primary stabilization, a gastroscopy was performed. The only significant finding during gastroscopy was an unusual regular, oval mucosal lesion in the distal esophagus, 7–8 mm in size, with a small adherent clot, without active bleeding (Figure 1A,B). Afterwards, the patient was intensively monitored, and high‐dose parenteral proton pump inhibitors were administered. During observation, the vitals remained stable, the control blood count performed several hours later was slightly reduced (Hb, 129 g/L; RBC, 4.20 × 10^12^; HCT, 0.35; WBC, 6 × 10^9^; PLT, 347 × 10^9^) and the patient insisted on intense back pain. A dynamic CT of the thorax and abdomen was performed. The CT scan revealed dilated thoracoabdominal aorta through her whole extension. The aneurysmatic dilation of the distal thoracic aorta at the level of the diaphragm was 6.3 cm in size, with irregular flow lumen and presence of mural and eccentric periaortic inflammatory thrombotic masses. The dilation continued towards the abdominal aorta, reaching 4 cm in size at the suprarenal level, 3.5 cm in the infrarenal segment, and propagated towards the common iliac arteries. The thrombotic mass occluded the branching lumen of the celiac trunk, without affecting the branching of the splenic and hepatic artery. At the level of the diaphragm, on the ventrolateral segment of the circumference, an active contrast extravasation in the periaortic area was registered, with periaortic fat stranding, and air inclusions in the mediolateral segment of the extravasation. Inferiorly, in the distal esophagus, above the gastroesophageal junction there was no clear demarcation between the fat tissue and the distal esophagus, indicating a formation of an AEF (Figure 2). After surgical consultation, an urgent open surgery was performed at the University Clinic for Cardiac Surgery. The aortic aneurysm was opened, the thrombotic masses were removed and the esophageal defect was repaired. The aortic reconstruction was performed by using a 22 mm tubular graft, which was proximally anastomosed with the thoracic aorta and distally with the aortic bifurcation. By using 5 and 6 mm Dacron grafts, anastomoses between the tubular graft and the celiac trunk, the superior mesenteric artery and the renal arteries, were made.

## 4 CONCLUSION AND RESULTS (OUTCOME AND FOLLOW‐UP)

Shortly after the patient was withdrawn from the extracorporeal circulation, a severe hemodynamic instability with persistent hypotension and bradycardia occurred followed by a lethal outcome despite the administration of high‐dose inotropes and vasopressors.

## 5 DISCUSSION

Although there are several similar cases previously reported, still, there are few unusual and specific aspects of our case that should be discussed in more details. Our patient did not report any previous trauma, cardiovascular, gastrointestinal, or malignant diseases. Also, lacking the typical clinical triad, the natural course was not typical. Although the proportion of AEF that presents with Chiari's triad is not that high,^4^ still, most patients present with hematemesis, chest pain, and a significant circulatory instability. Our patient was clinically and circulatory stable, he complained of back pain and the bleeding was not particularly massive. Hence, at presentation, there were not many elements that would reasonably rise the clinical suspicion for the presence of this entity.

Regarding the endoscopic appearance of the lesion representing the aortoesophageal communication, there is a large variability in the endoscopic aspect among different reports. Most authors report fresh blood into the gastrointestinal lumen and a lesion described as an ulcer or a mucosal defect with an adherent clot or a fibrin exudate.^1^, ^9^, ^10^, ^11^ A livid mucosa due to submucosal hematoma or a protruding pulsative luminal mass with narrowing of the esophageal lumen has also been described.^6^, ^12^ Interestingly, the initial endoscopy often cannot detect the culprit bleeding lesion, and according to the literature, the AEFs were detected on time only in approximately 25% of the cases.^4^ Although not diagnostic, the presence of fresh blood, the localization and the endoscopic aspect of the lesion in our patient was indicative of a communication between the digestive tract and a vascular structure. Still, the continuous back pain obliged us to perform additional diagnostic imaging and to seek for a specific and unusual pathology.

The CT scan is the most useful diagnostic tool in the diagnosis of AEF. Although, it does not always clearly identify the actual fistulous tract, the CT scan can demark the aneurysmatic sac, the thrombotic mass, and the mural thickening of the esophageal wall. Most importantly, it can detect the air bubbles in the thrombotic mass, a finding that is pathognomonic for the diagnosis of AEF.^1^, ^2^, ^7^, ^12^ The performed CT revealed the definitive diagnosis in our patient. However, it is rather unusual that the active contrast extravasation was detected in the abdomen, closely below the diaphragm, while the invasion in the gastrointestinal tract was located in the distal esophagus. That indicates a development of a complex abdominal aneurysm affecting a long aortic segment complicated with an inflammatory thrombotic process invading the esophageal wall.

UGB related to AEF is a potentially fatal condition associated with a high mortality rate that reaches 77% if treated and 100% without intervention.^5^ The therapeutic approach depends on a patient's current hemodynamic stability and general clinical state. However, successful management of AEF is rare.^2^ There are two main available treatment options: open surgery and thoracic endovascular aortic repair (TEVAR). Despite treatment, the conventional surgical repair reports in‐hospital mortality rate that approaches 40%.^13^ TEVAR is an innovative, safe, and less invasive technique associated with lower mortality and morbidity. It is the preferred alternative of open surgery, especially in the emergency setting and in cases with hemodynamic instability.^14^, ^15^, ^16^, ^17^, ^18^ However, TEVAR does not definitely repair the esophagus, and there is an increased risk of mediastinitis and infection of the stent‐graft.^13^ Therefore, TEVAR should only be used as a “bridge” to a definitive surgical intervention,^15^ except in patients with cancer‐related AEF.^2^ Alternatively, in order to control the massive bleeding, temporizing measures such as Sengstaken–Blakemore tube placement and radiologic embolization have been occasionally implemented in the past.^19^, ^20^, ^21^ In patients with AEF, initial management with TEVAR followed by an open surgical repair along with appropriate antibiotic use, is associated with a lower mortality rate and better outcomes and is currently the preferred therapeutic approach.^15^ However, despite the better short‐time survival after endovascular repair, still, both procedures are associated with a similar long‐term mortality.^22^

In North Macedonia, approximately 10 TEVAR procedures are performed yearly and all of them are planned and performed electively. Considering the small number of performed interventions, the price, and the different available sizes of the stent graft, it is not possible to obtain a stent graft on demand and to perform the TEVAR procedure in an urgent clinical setting. Additionally, considering the remarkable size and morphology of the aneurism that extended up to the common iliac arteries, according to the expert opinion of the vascular surgeon, performing the TEVAR procedure in this particular case would have been challenging. Hence, taking into account the available therapeutic options in our professional environment, open surgical repair was the only available treatment option in our case. However, due to significant hemodynamic instability, shortly after the vascular and digestive repair was performed, a lethal outcome occurred.

Despite the rapid diagnostic process, the accurate diagnosis, and the appropriate hemodynamic support provided by a multidisciplinary approach, unfortunately, in the end, the patient did not survive. We are aware of the fact that case studies presenting rare conditions with an odd presentation as ours, may sometimes be susceptible to publication bias. However, despite the unfavorable outcome, we feel that the benefit of raising awareness for this entity by the publication of the article would overcome the harm of the potential publication bias.

## 6 CONCLUSION

UGB related to AEF is a rare, but fatal life‐threatening condition in most reported cases. A high index of suspicion and clinical awareness, early recognition, prompt medical intervention, good emergency services, and interdisciplinary collaboration could contribute to early diagnosis and increase the probability for better outcomes.

## AUTHOR CONTRIBUTIONS

**Elena Curakova Ristovska:** Writing – review and editing. **Gregor Krstevski:** Conceptualization; writing – original draft. **Misel Andov:** Resources. **Aleksandar Kolev:** Conceptualization. **Kristijan Bundovski:** Visualization. **Kemal Rusiti:** Resources. **Svetozar Antovic:** Supervision. **Ivan Rankovic:** Validation. **Smiljana Bundovska Kocev:** Investigation. **Natasa Hadzi‐Nikolova Alcinova:** Investigation. **Ante Bogut:** Visualization.

## FUNDING INFORMATION

There was no specific funding for this article.

## CONFLICT OF INTEREST STATEMENT

The author declares that there are no conflicts of interest regarding the publication of this paper.

## CONSENT

Written inform consent was obtained from the patient to publish this report in accordance with the journal's patient consent policy.

## Data Availability

The data used to support this study are included within the article.
